# Buerger Disease: Pathological Changes in Elderly Patients

**DOI:** 10.3400/avd.oa.21-00142

**Published:** 2022-03-25

**Authors:** Takehisa Iwai, Hiroko Kume, Shinya Koizumi, Kenichi Sakurazawa, Kaori Honma, Hiroko Ogasawara, Tamiko Takemura, Mitsuhiro Kishino, Tomoko Kagayama

**Affiliations:** 1 Keiyukai Tsukuba Vascular Center, Moriya, Ibaraki, Japan; 2 NPO Buerger Disease Research Institute, Moriya, Ibaraki, Japan

**Keywords:** Buerger disease, thromboangiitis, aging, atherosclerotic risk factors, atherosclerosis

## Abstract

Reports of vascular lesion changes in elderly Buerger disease patients are rare. Patients are expected to continue to have typical Buerger disease even after the age of 50. However, after 50, when patients suffer from atherosclerotic risk factors, such as hypertension, diabetes mellitus, or hyperlipidemia, what kind of changes will occur? We will report on 3 cases of hypertension, diabetes mellitus, or hyper lipidemia after or around 50 years of age. As a result, atherosclerosis was present in the iliac or aortic regions in the remaining thromboangiitis lesions below the groin area. (This is secondary publication from the J Jpn Coll Angiol 2021; 61: 107–113.)

## Introduction

The number of patients with Buerger’s disease in Japan is steadily decreasing.^2–4)^ One of the causes seems to be related to the complexity of the diagnostic criteria for Buerger’s disease. Furthermore, from the results of our research, we believe that smoking cessation and the spread of oral care are largely involved in the changes.^5,6)^ We encountered 34 new patients with Buerger’s disease in 11 years.^7)^

This time, we examined three patients with Buerger’s disease (2 males, 71 and 82 years of age; 1 female, 74 years of age) who were not treated despite additional atherosclerosis-risk factors after the age of 50. At the time of onset before the age of 50, the diagnostic criteria of Shionoya were completely satisfied (**Table 1**).^8)^ In addition, it was compared with seven elderly patients in their 70s who had no risk factors.

**Table 1:** Table 1 Clinical diagnostic criteria (from Shionoya^8)^)

1	Smoking history
2	Onset before the age of 50 years
3	Infrapopliteal arterial occlusive lesions
4	Either upper limb involvement or phlebitis migrans
5	Absence of atherosclerotic risk factors other than smoking

## Materials and Methods

Among the patients who experienced Buerger’s disease between 2007 and 2020, those who had hypertension, hyperlipidemia, and diabetes after the age of 50 were diagnosed with atherosclerosis based on surgical or diagnostic procedures. The three examples were targeted. The course of these three cases is as follows.

**Figure 1:**
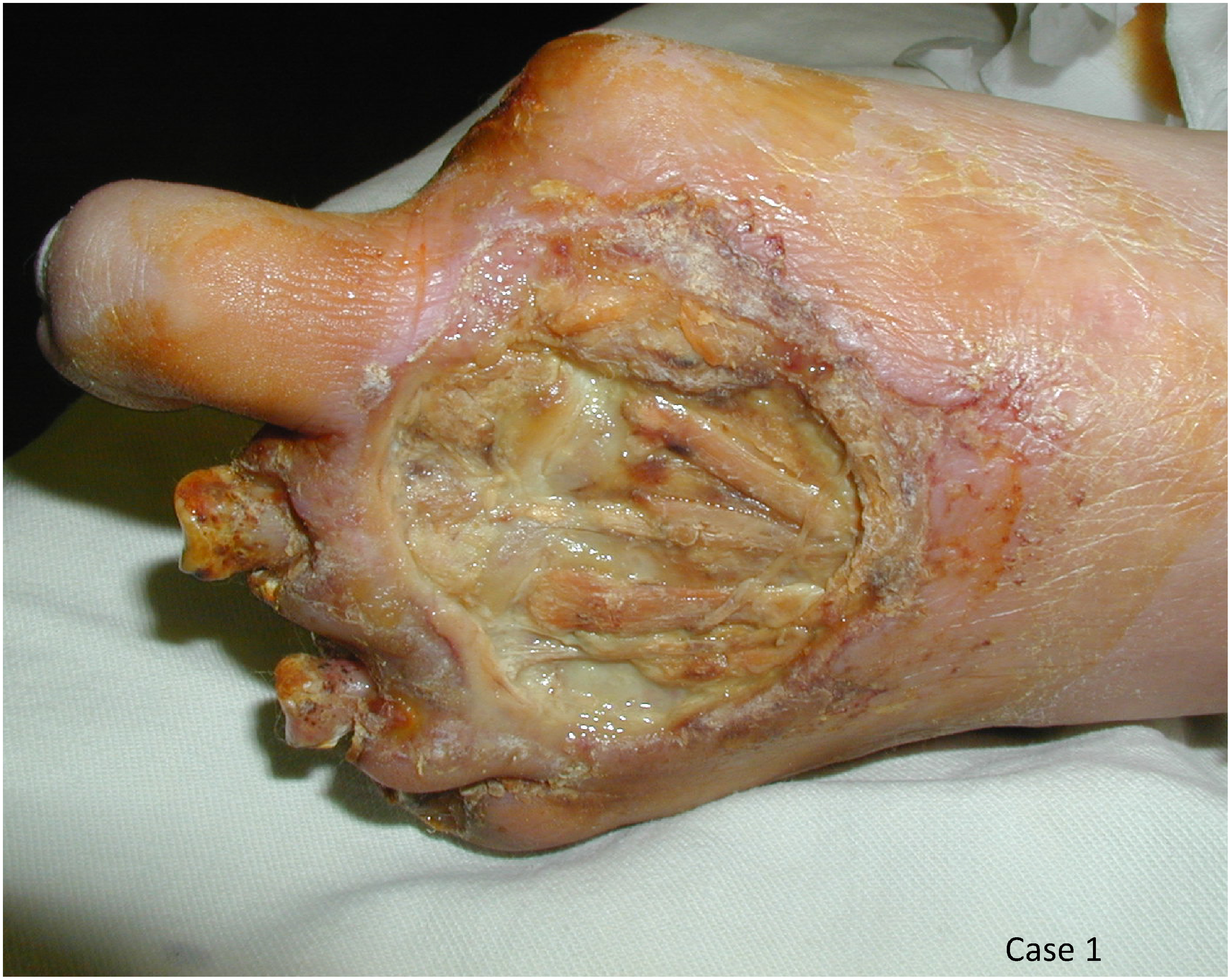
Fig. 1 Preoperative view of the left foot in Case 1.

**Figure 2:**
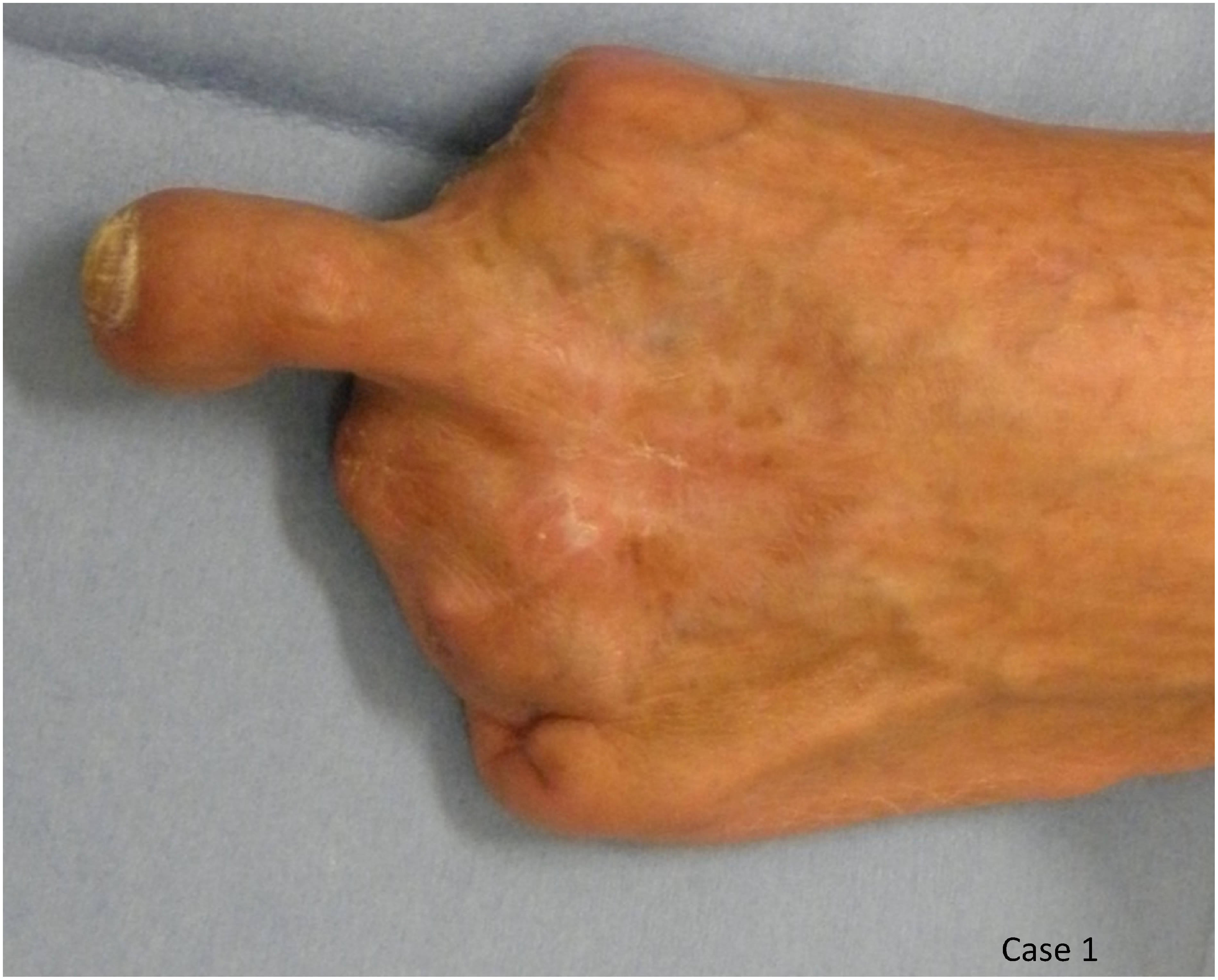
Fig. 2 Several months after the operation. Patient can walk without any pain.

**Figure 3:**
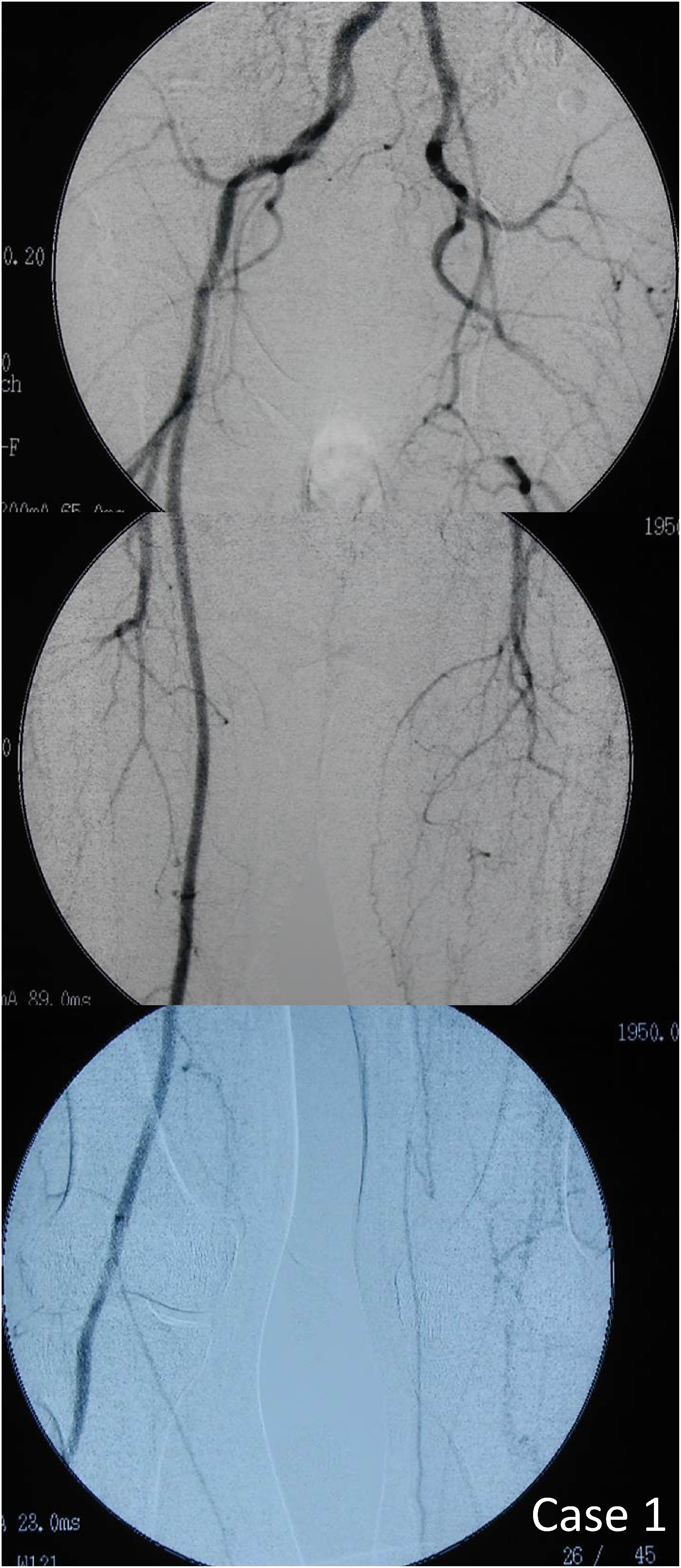
Fig. 3 Preoperative angiography of Case 1. The left external iliac artery occlusion and patent profunda femoris artery are seen. Below the groin, the left main arteries are occluded showing typical Buerger disease angiogram.

Case 1. A 71-year-old man was diagnosed with claudication in the left foot when he was 32 years of age. In addition, the heel and his first toe progressed to rest pain (severe pain). At the age of 35, he had an ulcer on his first toe, which was then amputated. He then had repeated minor amputations of his left toes from around the age of 50 (**Fig. 1**). He had a history of smoking 30 cigarettes/day for 30 years, has hypertension (150–160/80 mmHg) without medication, and diabetes or prediabetes (HbA1c about 7.1% from 6.7 and a fasting blood glucose level of 120 mg/dl), which progressed without medication from around 50 years old. At the age of 51, a bypass of the left common iliac artery to the profunda femoris artery was performed, and the percutaneous oxygen partial pressure was normalized, the left foot ulcer healed, and normal walking became possible (**Fig. 2**). Arteriography showed the left external iliac artery occlusion (not observed at age 32) and typical Buerger’s disease arterial findings below the inguinal ligament (**Fig. 3**). There are three missing teeth, but it is said that he had repeated bleeding when brushing his teeth.

**Figure 4:**
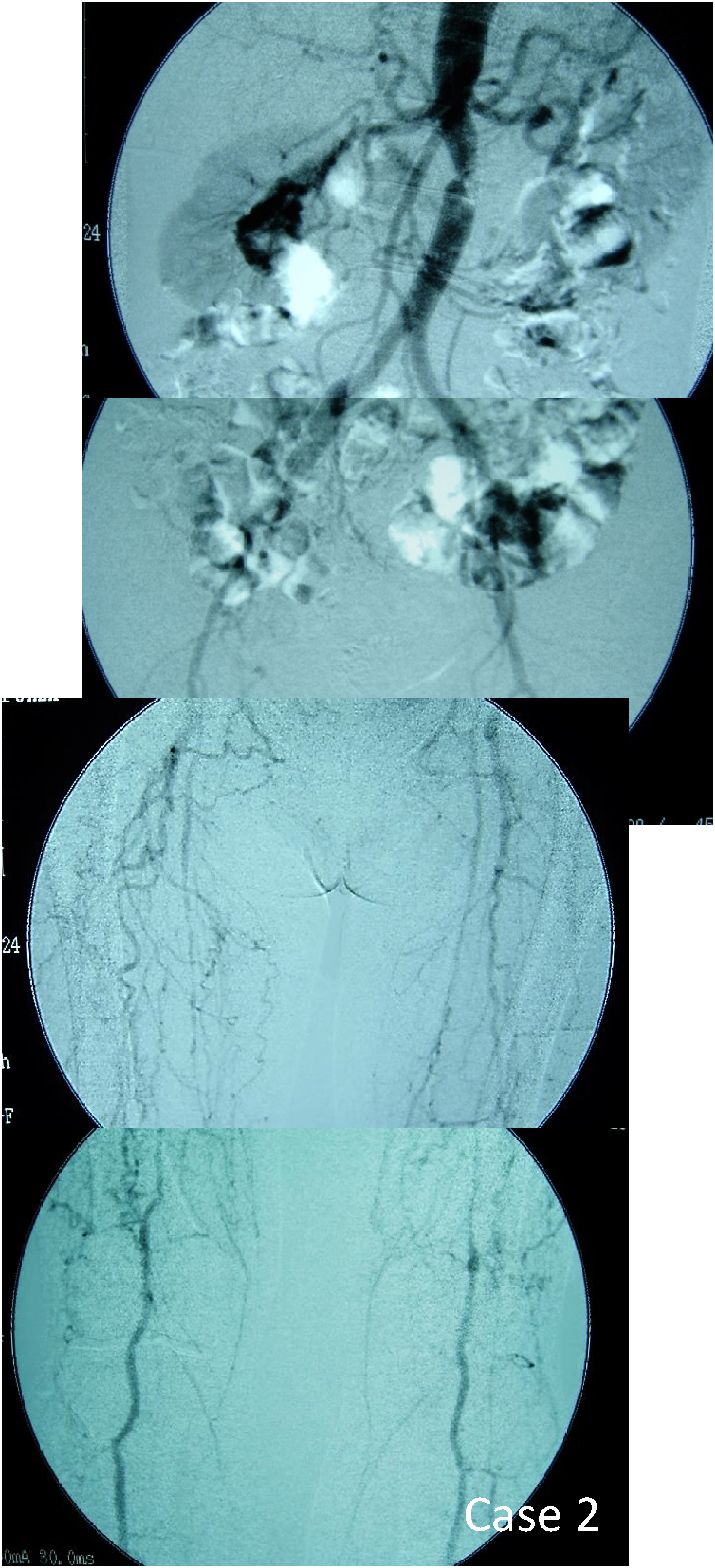
Fig. 4 Case 2. This shows the severe aortic stenosis and typical arterial occlusions and collateral systems of Buerger disease below the groin.

Case 2. A 74-year-old woman was diagnosed with Buerger’s disease when intermittent claudication appeared around the age of 40. When the medication was discontinued, rest pain appeared at the age of 56, and close examination revealed severe stenosis of the aorta in addition to the Buerger’s disease lesions below the inguinal ligament (**Fig. 4**). She received an aorto-bi-common iliac arterial bypass at the age of 58, and symptoms were relieved. The Ankle Brachial Pressure Index (ABI) is about 0.54 on both sides, but it does not interfere with daily life. She was diagnosed with hyperlipidemia (dyslipidemia, low density lipoprotein cholesterol (LDL): 150–140 mg/dl) after the age of 50 and started treatment with ethyl icosapentate around the age of 60. She smoked 5 cigarettes a day from around 30 years old and smoked for 15 years. She was diagnosed with severe chronic periodontitis because she had many missing teeth, and the periodontal pockets of her remaining teeth were as deep as 4–5 mm.

**Figure 5:**
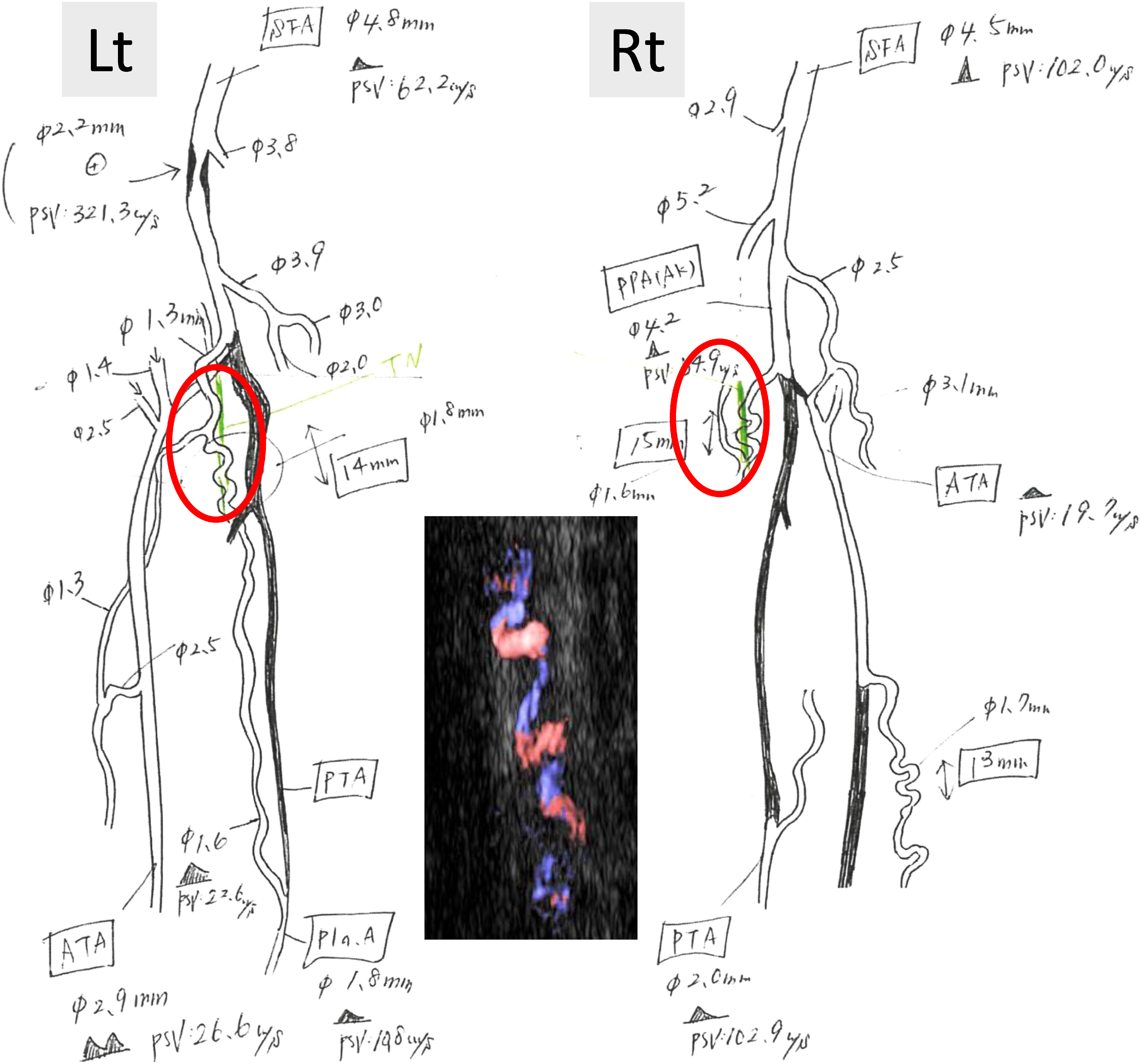
Fig. 5 Case 3. Below the knee, echo gram shows posterior tibial or anterior tibial arterial occlusions. Cork screw collateral are recognized along with the nerve (middle).

Case 3. An 82-year-old man who, at the age of 34, felt coldness and claudication in the upper and lower limbs, which gradually worsened. From around the age of 40, his fingers and toes became cold and numb during rest, and he was diagnosed with Buerger’s disease; he then underwent vascular surgery at the age of 45. Angiography showed typical Buerger’s disease findings in the lower extremities. The recent (2021) echo findings of the lower limbs and the corkscrew-like collateral, coiled with the nerve (**Fig. 5**). He had abdominal angina symptoms, had celiac artery occlusion, and superior mesenteric artery stenosis. At the age of 46, he underwent abdominal aorta-common hepatic artery bypass and lumbar sympathectomy. Symptoms subsided with smoking cessation and antidiarrheal agents. He started smoking during his teens and continued for 50 years with 10 pieces/day. He lost all his teeth at the age of 40. He had hyperlipidemia (LDL: 155 mg/dl) for more than 10 years and only started statin medication 3 years ago. The recent ABI is as follows: right 0.45, left 0.58, Toe Brachial Pressure Index right 0.3, left 0.31. During this period, the lesions below the inguinal region have hardly changed.

The common points among the above three cases were that all of them received antiplatelet agents or small doses of aspirin. No significant findings were found in Elektrokardiogramm, echocardiography, or carotid echogram for coronary artery or cerebrovascular disorders. Computed tomography (CT), coronary angiography and magnetic resonance angiography were not performed in all cases.

On the other hand, 7 patients aged 70 years or older who did not develop atherosclerosis-risk factors after the age of 50 (all males, average 76.4 years old; 2 had hypertension from around 50 years old but neglected the risk factors because they were well-controlled). The presence or absence of atherosclerosis of the aorto-iliac and femoral arteries was examined by enhanced CT, echography, and auscultation/palpation of the abdomen and inguinal regions.

This study has been approved by the Keiyukai Ethics Committee (No. R03-01).

This study was conducted in accordance with the Declaration of Helsinki.

## Results

**Figure 6:**
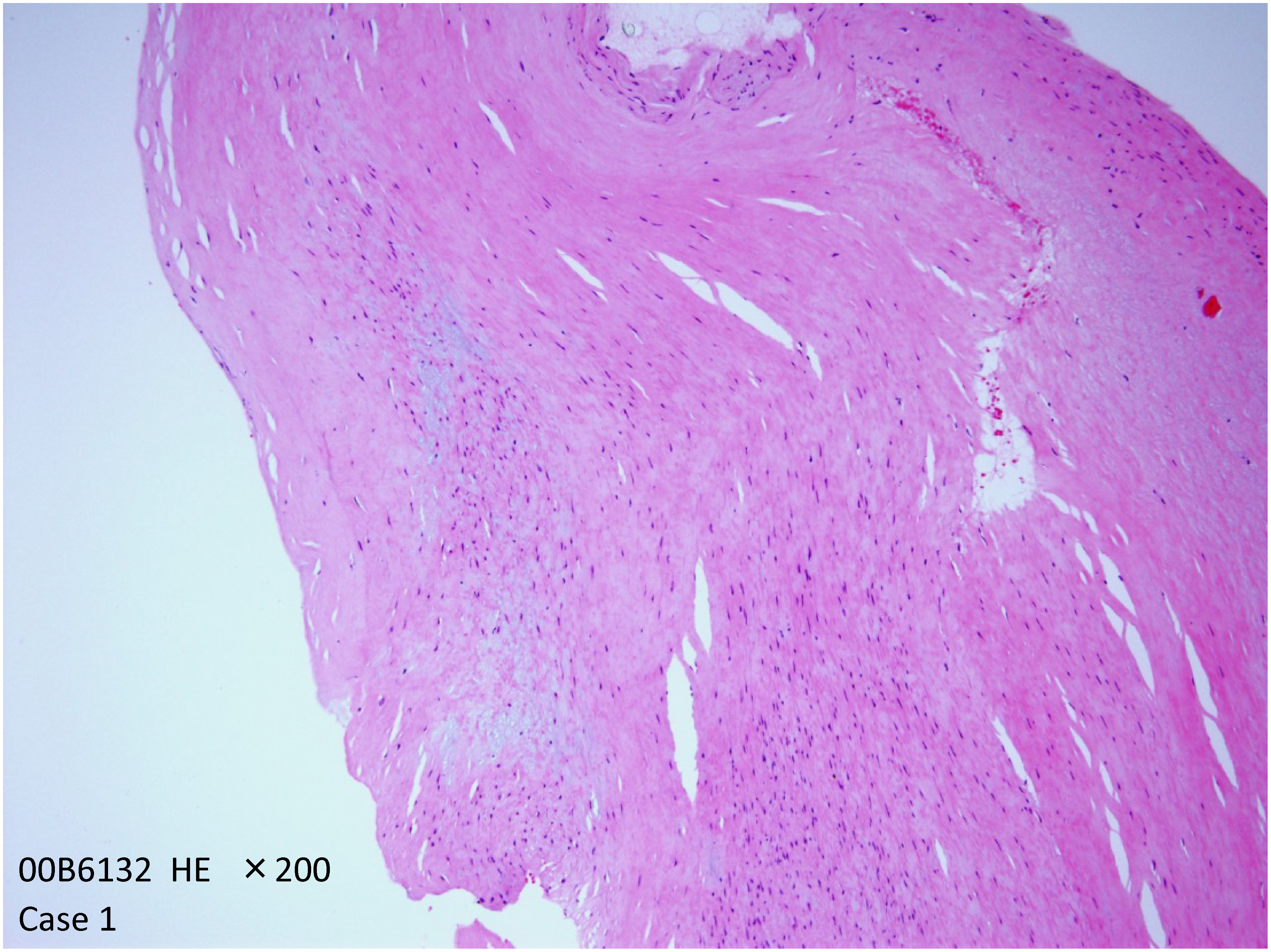
Fig. 6 Case 1. HE stain shows typical atherosclerosis.

**Figure 7:**
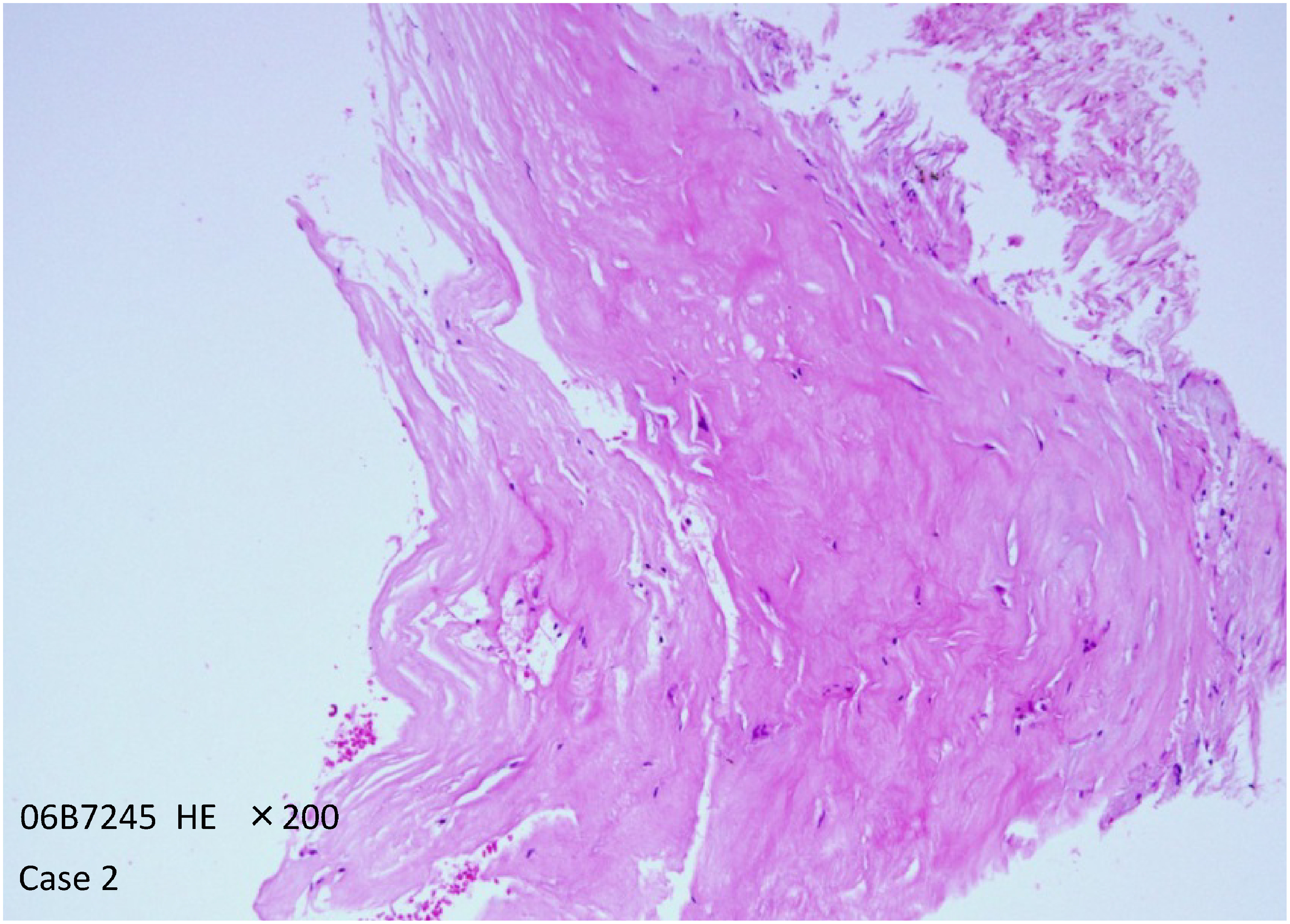
Fig. 7 Case 2. This specimen shows calcification and typical atherosclerosis changes.

**Figure 8:**
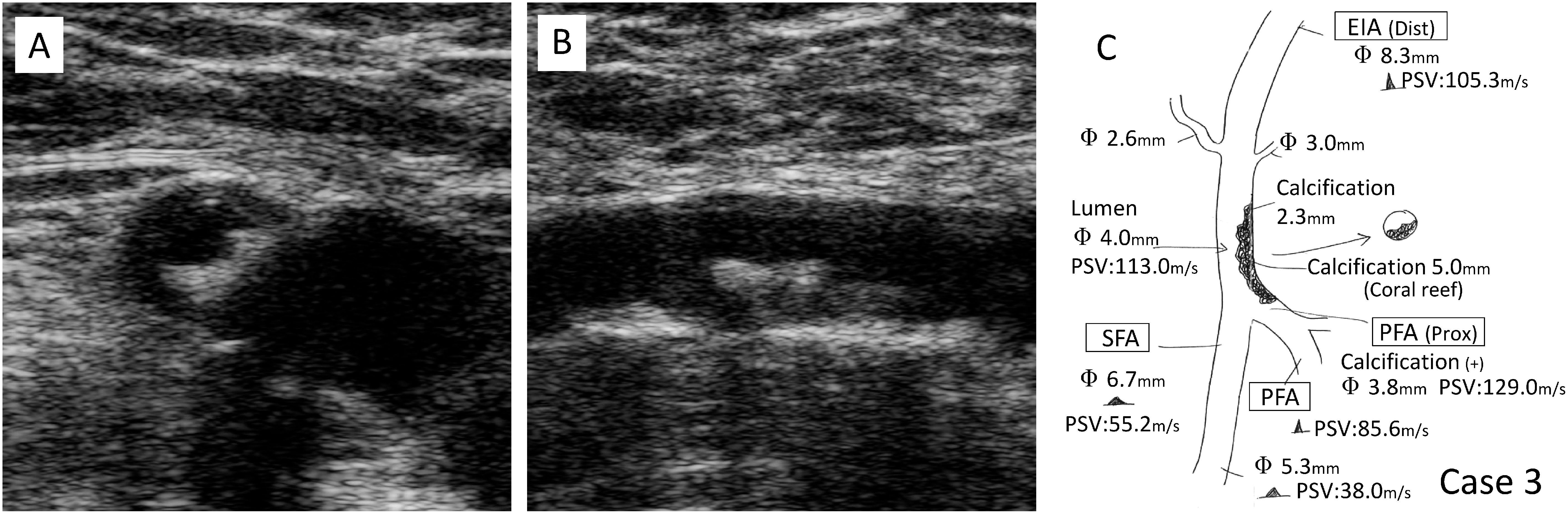
Fig. 8 Case 3. In the right common femoral artery, typical coral reef atheroma is seen.

Hematoxylin and Eosin staining of the iliac artery fragment collected at the time of surgery in Case 1 (**Fig. 6**) showed atherosclerotic changes with a high degree of fibrous thickening, and it was judged that the external iliac artery occlusion was due to atherosclerosis. Atherosclerosis collected from the abdominal aorta in Case 2 was diagnosed as atherosclerosis with strong calcification and atheroma (report from the Department of Pathology, University Hospital, both of which underwent surgery) (**Fig. 7**). In case 3, arterial echography showed coral reef-like atheroma deposits in the right common femoral artery (**Fig. 8**).

In the 7 patients who did not develop the risk factors, there was calcification in the aorto-iliac/femoral arterial region, but no atheroma (arteriosclerosis obliterans) that caused stenosis or occlusion was observed. Carotid echography also showed no findings suggestive of atherosclerosis without bruit.

## Discussion

**Figure 9:**
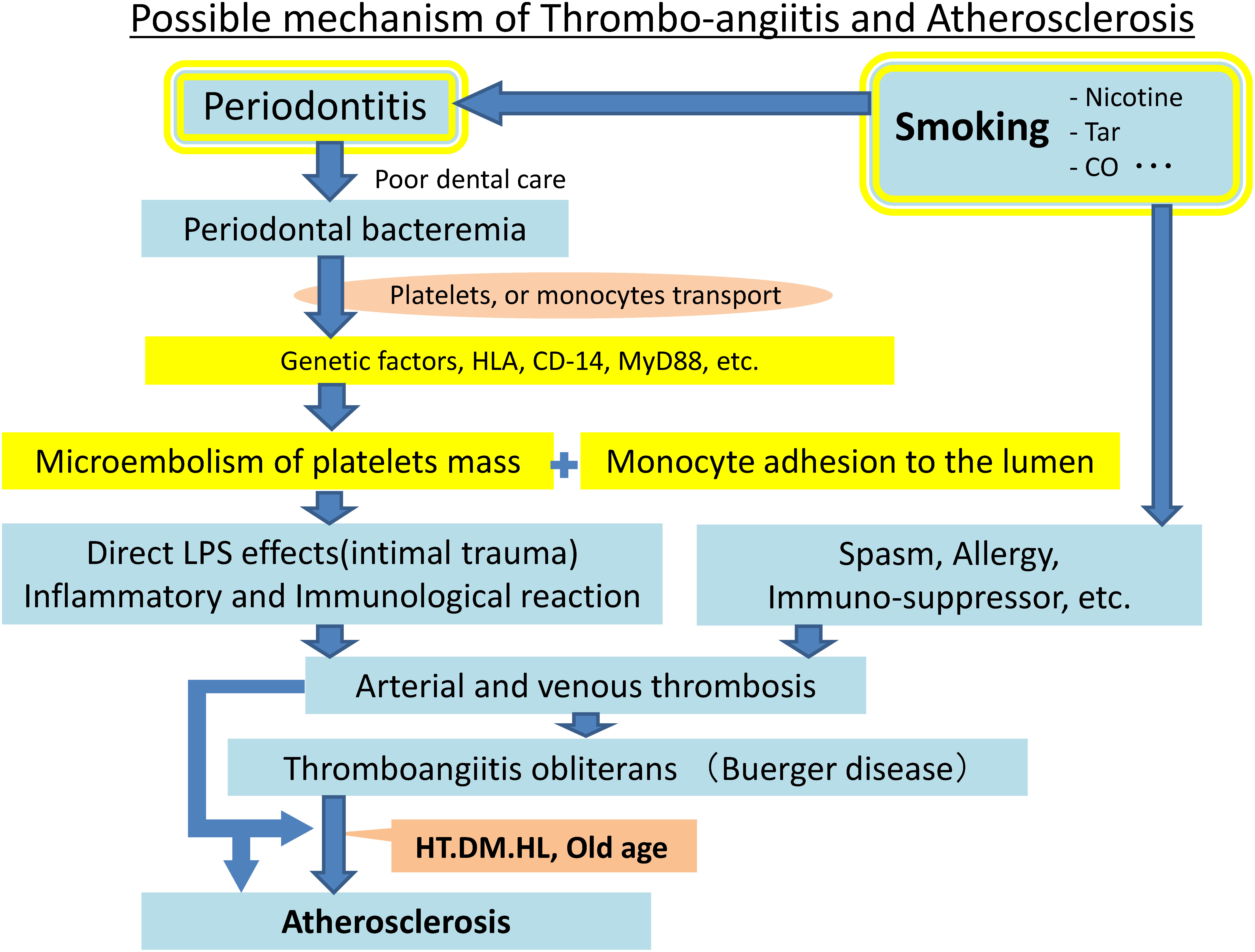
Fig. 9 Iwai’s proposal of pathogenesis in Buerger disease and athrosclerosis.^6)^

**Figure 10:**
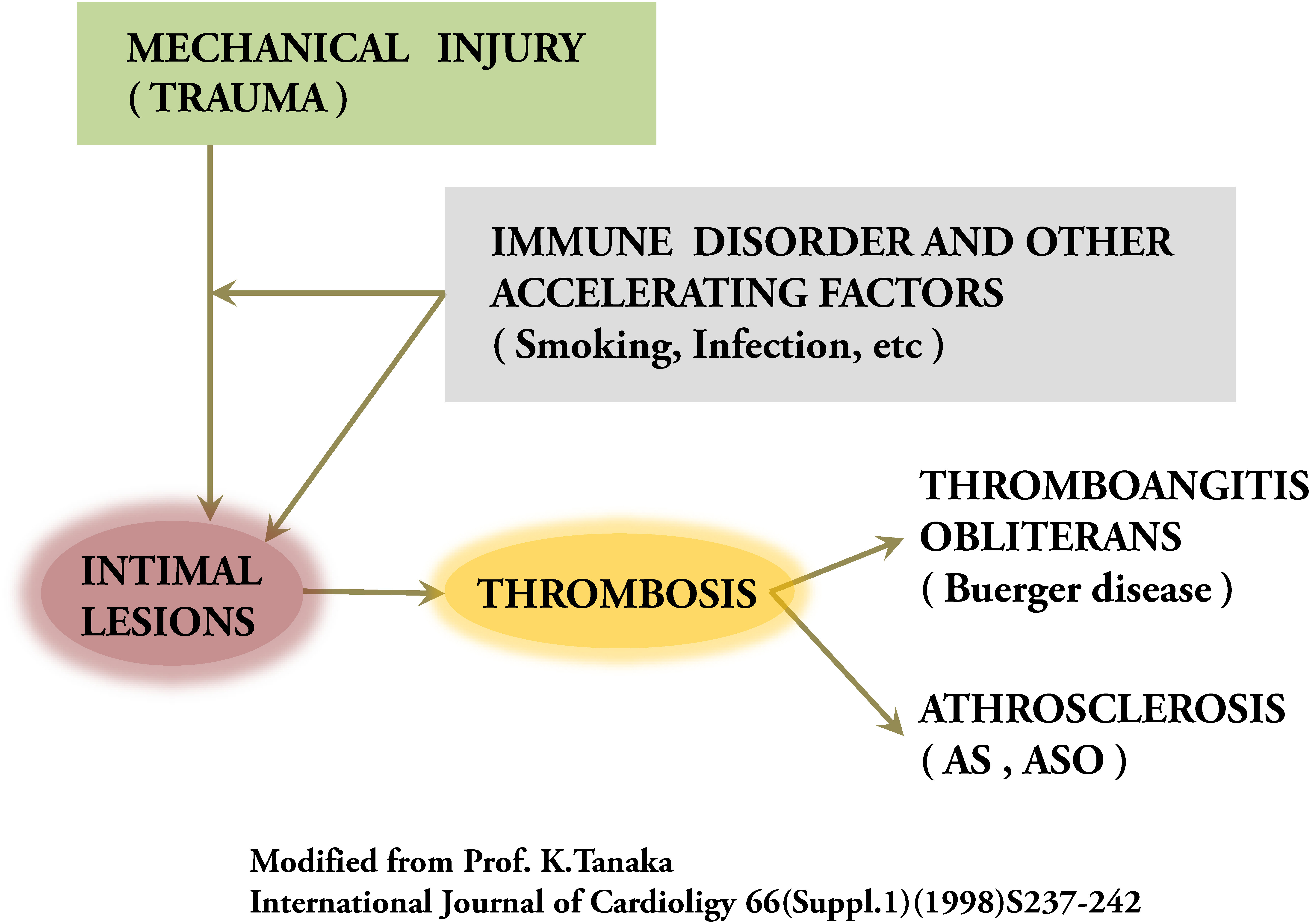
Fig. 10 Tanaka’s proposal of etiological mechanisms in Buerger disease and athrosclerosis.^9)^

This paper is a clinical study investigating why atherosclerosis overlaps in elderly people with Buerger’s disease. **Fig. 9** shows the developmental process of Buerger’s disease and atherosclerosis, which was cited from a recently published paper.^6)^ Furthermore, **Fig. 10** shows the hypothesis (partially modified) of the development of Buerger’s disease and atherosclerosis presented by Professor Kenzo Tanaka in 1998.^9)^ Both diseases are similar or marginal diseases that are on the same line. As far as Buerger’s disease is concerned, periodontal bacteria were identified by PCR from human vascular materials, thrombus was created by puncturing disease-free mice vessels using the same periodontal bacteria, and the pathological materials were very similar to each stage of Buerger’s disease pathology.^5)^ It is believed that the success of the experimental study satisfied Koch’s three postulates, and further immunological studies proved that it was innate immunity, thus satisfying Koch’s four postulates.^6)^ If this developmental process is correctly recognized, atherosclerosis should develop newly in addition to the vascular changes in Buerger’s disease if the patients with Buerger’s disease are affected by the factors promoting atherosclerosis and aging.^6,9)^ As evidence of this, the three cases introduced here were able to show pathological specimens in two cases and typical echo findings in one case. On the other hand, we examined 7 patients with Buerger’s disease (all males) who did not develop atherosclerosis risk factors even after they were over 50 years old and in their 70s, but there was no evidence of complications of atherosclerosis with clear atheroma.

Furthermore, when the frame of Buerger’s disease is removed, it can be understood from **Fig. 9** that there is a route in which periodontal disease causes bacteremia and atherosclerosis appears when any factors of promoting atherosclerosis is added. Ohneda et al.^10)^ introduced cases of a coexistence of Buerger’s disease and atherosclerosis from his autopsy cases in the elderly group, but he did not study up to the pathogenesis bases. From the history of Buerger’s disease research to date, only a very limited number of people are diagnosed with Buerger’s disease who have a history of heavy smoking, no hypertension, no diabetes, and no hyperlipidemia, which were associated with limb arterial occlusion and phlebitis migrans. This is a very strict diagnostic criterion for the diagnosis of Buerger’s disease (**Table 1**).^8)^ In this era, “true” Buerger’s disease will decrease, and “Buerger’s disease subtypes” that do not match with the criteria for the diagnoses of Buerger’s disease will increase. At present, Buerger’s disease is properly named if the patient is under the age of 30, but when even one risk factor is added after that, it will no longer be “true” Buerger’s disease and it will not match the diagnostic criteria and will be regarded as a “subtype of Buerger’s disease” or called as atherosclerosis. Diabetes mellitus, in particular, is often confused with the arterial occlusion of the distal arteries below the knee area.^11)^ Therefore, in order to make the diagnosis easier to understand, if the patient has one of the three risk factors, the occlusive change should be called atherosclerosis (or arteriosclerosis obliterans) regardless of age. It is called “juvenile” type in the young and becomes “juvenile atherosclerosis.” In fact, when accredited as an intractable disease designated by the Ministry of Health, Labor and Welfare, the strict diagnostic criteria that are subject to document screening may be one of the factors that reduce Buerger disease mumbers. It is interesting to note that the age limit in Japan is 50 years old, but the limit is 45 years old in some countries.^12)^

It is impossible to diagnose Buerger’s disease from the causative organism. There are numerous papers stating that atherosclerosis is also associated with periodontal bacteria and other oral bacteria,^13–15)^ and the delicate difference between the two major occlusive arterial diseases, namely Buerger disease and atherosclerosis, is as shown in this paper. Namely, this is because it can be said that there are risk factors or not. Again, this is because Buerger disease’s is the pathogenetic condition in the absence of the risk factors, and the pathogenetic condition in which the risk factors are added is atherosclerosis. To summarize, it is Buerger’s disease that occurs in heavy smokers without hypertension, hyperlipidemia, and diabetes, and if there is even one of them, it should be Buerger’s disease plus atherosclerosis or simply atherosclerosis. The age limitation is a stumbling block. Please refer to the recent paper^6)^ for the process of reaching that conclusion. Looking at the process of research on Buerger’s disease in Japan, it is stated that the proximal changes in the iliac arteries or the aorta seen in Buerger’s disease are described as the progression of secondary thrombi.^16)^

In the 1950s, there was a “Buerger disease exclusive movement” that Buerger disease was juvenile atherosclerosis. This is because atherosclerotic lesions were found one after another, especially on the proximal region, from patients with Buerger’s disease.^17,18)^ This controversy was settled by showing cases of young people, and cases of Japan and South Korea that smoking cessation resulted in the complete relief of Buerger’s disease.^19)^ However, some doctors still do not believe in the existence of Buerger’s disease ever since.^1)^ The diagnostic criteria are narrow, and in addition to the age limit, there is a special restriction on diagnosis, such as risk factors, and Buerger’s disease rarely exists.

Platelet clots and monocytes containing periodontal bacteria are scattered throughout the body, and due to the characteristics of Gram-negative anaerobic bacteria, the bacteria die immediately, lipopolysaccharide is released, and endothelial cells are destroyed.^20,21)^ This is considered to be the beginning of the two arterial occlusive diseases. Dead bacteria become an inflammation core,^5)^ and it is thought that arterial occlusion progresses from the tip of the limbs where these substances are not processed well or cannot be processed. Periodontal bacteria have been found in occluded blood vessels throughout the body, but most are asymptomatic.^22,23)^ In coronary artery occlusion, *Aggregatibacter actinomycetemcomitans* (Aa), a bacterium with extremely strong arterial destructive power, has been found. The strength of the destructive power of Aa bacteria was introduced in recent literature as an animal model.^6)^ In addition, the degree of periodontal disease has been a list of clinical findings so far, and it was unstable to grasp the degree, but in recent years, a scoring system has been promoted, and there is a possibility that a correlation with medical research will make it easier to understand.^24)^

*Chlamydia pneumoniae*, cytomegalovirus, *Helicobacter pylori*, among others,^25)^ have been found to enter the blood vessels, but they may be supporting the viciousness of periodontal disease bacteria, but from the types of bacteria or virus, I think that periodontal bacteria will be overwhelming in terms of bacterial numbers and so on.

## Conclusion

From the pathological materials and echogram findings of elderly patients with Buerger’s disease, it was possible to explain that atherosclerosis progresses when atherosclerosis-risk factors are added after the age of 50, while leaving the typical angiographic findings of Buerger’s disease. There is a need to make some improvements or changes to the very narrow diagnostic criteria for Buerger’s disease.

## Notes

This is secondary publication from the J Jpn Coll Angiol 2021; 61: 107–113.
